# Impact of routine PCV7 (Prevenar) vaccination of infants on the clinical and economic burden of pneumococcal disease in Malaysia

**DOI:** 10.1186/1471-2334-11-248

**Published:** 2011-09-21

**Authors:** Syed Aljunid, Gulifeiya Abuduxike, Zafar Ahmed, Saperi Sulong, Amrizal Muhd Nur, Adrian Goh

**Affiliations:** 1 United Nations University-International Institute For Global Health, Kuala Lumpur, Malaysia; 2 International Case-Mix and Clinical Coding Centre, UKM Medical Centre, Faculty of Medicine, University Kebangsaan Malaysia, Kuala Lumpur, Malaysia; 3 Azmi Burhani Consulting, Kelana Jaya, Malaysia

## Abstract

**Background:**

Pneumococcal disease is the leading cause of vaccine-preventable death in children younger than 5 years of age worldwide. The World Health Organization recommends pneumococcal conjugate vaccine as a priority for inclusion into national childhood immunization programmes. Pneumococcal vaccine has yet to be included as part of the national vaccination programme in Malaysia although it has been available in the country since 2005. This study sought to estimate the disease burden of pneumococcal disease in Malaysia and to assess the cost effectiveness of routine infant vaccination with PCV7.

**Methods:**

A decision model was adapted taking into consideration prevalence, disease burden, treatment costs and outcomes for pneumococcal disease severe enough to result in a hospital admission. Disease burden were estimated from the medical records of 6 hospitals. Where local data was unavailable, model inputs were obtained from international and regional studies and from focus group discussions. The model incorporated the effects of herd protection on the unvaccinated adult population.

**Results:**

At current vaccine prices, PCV7 vaccination of 90% of a hypothetical 550,000 birth cohort would incur costs of RM 439.6 million (US$128 million). Over a 10 year time horizon, vaccination would reduce episodes of pneumococcal hospitalisation by 9,585 cases to 73,845 hospitalisations with cost savings of RM 37.5 million (US$10.9 million) to the health system with 11,422.5 life years saved at a cost effectiveness ratio of RM 35,196 (US$10,261) per life year gained.

**Conclusions:**

PCV7 vaccination of infants is expected to be cost-effective for Malaysia with an incremental cost per life year gained of RM 35,196 (US$10,261). This is well below the WHO's threshold for cost effectiveness of public health interventions in Malaysia of RM 71,761 (US$20,922).

## Background

*Streptococcus pneumoniae *(*S. pneumoniae*) is a bacterial pathogen responsible for significant morbidity and mortality worldwide, particularly in young children and the elderly [1-4]. Non-invasive pneumococcal disease is caused by *S. pneumoniae *infection of mucosal tissue, such as the upper respiratory tract, middle ear and sinuses which can lead to non-bacteraemic pneumonia, acute otitis media (OM) and sinusitis. The more severe invasive pneumococcal disease (IPD) occurs when bacteria disseminates into the bloodstream and central nervous system resulting in bacteraemia, meningitis, and bacteraemic pneumonia [5-8]. It is estimated that up to one million children under 5 years of age die every year from pneumococcal pneumonia, meningitis and sepsis [9,10]. Data from the World Health Organization (WHO) shows that pneumococcal disease is the leading cause of vaccine-preventable death in children younger than 5 years of age worldwide [11]. In 2009, pneumonia was the 4^th ^leading cause of death in Ministry of Health (MOH) Hospitals in Malaysia with 10.4% of hospital mortality while respiratory diseases were the 3^rd ^leading cause of hospitalisation with 9.4% of discharges [12].

Although antibiotic treatment for pneumococcal diseases are available, there are still unmet medical needs as reflected in the mortality and morbidity data. Prevention via vaccination is an important intervention that could reduce the incidence of pneumococcal infection and its complications [5]. One of the available vaccines in Malaysia at the time of this study was Prevenar^®^, a Heptavalent Pneumococcal Conjugate Vaccine (PCV7), indicated for the active immunization of infants and children from 6 weeks through 9 years of age. Prevenar contains saccharides of the capsular antigen of *S. pneumoniae *serotypes 4, 6B, 9V, 14, 18C, 19F and 23F individually conjugated to the diphtheria Cross Reactive Material 197 (CRM197) protein [13]. Following recommendation by the Strategic Advisory Group of Experts (SAGE) on immunization, the World Health Organization considers that the pneumococcal conjugate vaccine should be a priority for inclusion in national childhood immunization programmes [6]. As of January 2010, high pneumococcal vaccination coverage has been achieved in 43 countries as part of national immunization programmes or through other means [14]. PCV7 has been available in Malaysia since 2005 but has yet to be included in the national vaccination programme.

The herd effect, also known as herd immunity, is defined as the reduction of infection or disease in the unimmunised segment as a result of immunising a proportion of the population [15-17]. This effect is seen in vaccination with PCV7, where it has been shown to be able to reduce nasopharyngeal carriage of vaccine strains in immunised children, resulting in subsequent interruption of transmission to unimmunised contacts [18]. In countries where PCV7 has been included as part of the routine vaccination schedule, surveillance data has demonstrated that vaccination of specific populations not only provides immunised individuals with protection against pneumococcal disease, but also reduces the risk of developing pneumococcal illness in unimmunised individuals.. For example, in the United States, it is estimated that PCV7 has prevented more than twice as many cases of pneumococcal disease through indirect effects [19]. Another study from 8 states across the United States reported a 28% reduction in incidence of pneumococcal disease and a 55% reduction in conjugate vaccine serotypes in adults above 50 years of age after the introduction of PCV7 infant vaccination in year 2000 [20]. A study of households in Pennsylvania also found an 80% reduction (OR = 0.2, 95% CI 0.1-0.8) in the odds ratio of bacteraemic pneumococcal pneumonia among adults in households where young children had received PCV7 vaccination [21].

This study sought to estimate the disease burden of pneumococcal disease in Malaysia and to assess the cost effectiveness of routine infant vaccination with PCV7.

## Methods

A decision analytic model based on PCV7 immunization in the United Kingdom by McIntosh [22] was adapted to determine the effects and cost effectiveness of universal infant PCV7 vaccination compared with no vaccination for a hypothetical birth cohort of 550,000 infants over a 10-year time horizon. Similar to McIntosh, the model incorporated hospitalised cases of pneumococcal disease based on age-specific disease incidence rates. These included pneumococcal meningitis, pneumococcal septicaemia and pneumococcal pneumonia as well as a proportion of unspecified pneumonia and otitis media. All cases of pneumococcal infections requiring hospitalisation from pre-admission to post-admission follow-ups in the outpatient setting were included. Demographic assumptions within the model are described in Table 1. The analysis was performed from the payer perspective.

**Table 1:** Model assumptions on pneumococcal disease and vaccination effects

Parameter	Value	Source
**Population distribution**		
Birth cohort	550,000	[25,26]
20-39 years age group	8,770,937	[25,26]
40-64 years group	5,536,238	[25,26]
≥ 65 years group	1,051,371	[25,26]
Life expectancy at birth (years)	74.2	[30]
**PCV7 vaccine coverage**	495,000	90% of birth cohort [12]
**PCV7 vaccine compliance**	100%	
**Pneumococcal disease incidence**		
S. pneumonia isolated	100%	
Unspecified meningitis and bacteraemia	36%	[24]
All cause pneumonia	30%	[23]
**Paediatric fatality rate**		
Meningitis	0.2	[31]
Bacteraemia	0.1	[31]
All cause pneumonia	0%	[31]
Otitis media	0%	[31]
**Adult fatality rate**		
Meningitis	9.0%	[32]
Bacteraemia	4.6%	[32]
All cause pneumonia	0.17%	[32]
Otitis media	0%	[32]
**PCV7 serotype coverage**	66.7%	[28]
**PCV7 efficacy in infants**		
Pneumococcal meningitis	97.4%	[27]
Pneumococcal bacteraemia	97.4%	[27]
All cause pneumonia	6%	[27]
Otitis media	7%	[27]
**PCV7 efficacy decline over time (efficacy waning)**		
≤ 5 years post-vaccination	1% p.a.	[22]
6-10 years post-vaccination	3% p.a.	[22]
**Reduction on IPD in adults (herd effects)**		
20-39 years group	32%	[29]
40-64 years group	8%	[29]
≥ 65 years group	18%	[29]

This study was approved by the ethics committees of the National University of Malaysia (FF-12-2008) and the Ministry of Health Malaysia (NMRR-08-664-1819).

### Disease incidence estimation

The incidence of hospitalised pneumococcal disease was estimated from review of medical records from 2006-2007 at six tertiary hospitals. These six hospitals were selected to broadly represent Malaysia geographically as shown in Table 2.

**Table 2:** Primary sources of epidemiological data

Hospital	Type of Hospital	Region	Catchment population (% national population)
National University of Malaysia Medical Centre	Teaching hospital	Kuala Lumpur, Central Peninsula	603,692 (2.2%)
Hospital Tuanku Jaafar, Seremban	Ministry of Health	Seremban, Central Peninsula	311,898 (1.1%)
Hospital Universiti Sains Malaysia	Teaching hospital	Kota Bharu, Peninsula East Coast	341,232 (1.3%)
Hospital Tengku Ampuan Afzan, Kuantan	Ministry of Health	Kuantan, Peninsula East Coast	368,805 (1.4%)
Hospital Sultanah Bahiyah, Alor Setar	Ministry of Health	Alor Setar, Northern Peninsula	221,054 (0.8%)
Sarawak General Hospital	Ministry of Health	Kuching, East Malaysia	687,413 (2.5%)

Cases of hospitalised pneumococcal disease were primarily identified by International Statistical Classification of Diseases and Related Health Problems 10th Revision (ICD-10) classification at discharge. Additional patients positive for streptococcus pneumonia were also identified through review of laboratory records to ensure more comprehensive data capture. For the purpose of this study, ICD-10 pneumococcal disease codes included were: pneumococcal meningitis (code G00.1), pneumococcal septicaemia (code A40.3), and pneumonia due to *S. pneumonia *(code J13). In addition, the study identified other unspecified pneumonia (codes J15.9, J18, J18.9) and otitis media (codes H65, H66, H67). Thirty percent of all-cause pneumonia cases and 36% of meningitis and bacteraemia were attributed to *S. pneumoniae *[22-24]. All patient data was de-identified prior to analysis.

The incidence of pneumococcal disease by clinical presentation for children and adults in each hospital catchment area was calculated from epidemiological data from the six hospitals. Due to lack of any published estimates, assumptions about the population catchment and treatment provision by hospitals were obtained from a focus group discussion consisting of experienced clinicians and paramedics conducted in October 2008. The catchment population of UKM Medical Centre was assumed to be the entire population of the local district and 30% of the population in the neighbouring district. The catchment area for the remaining five hospitals was assumed to be the entire 2007 population of the urban area and 5% of the state population where the hospitals were located [25,26]. Each hospital was assumed to provide treatment for 50% of cases within its catchment area. The national incidence rate by age group and presentation in 2007 was estimated as the average disease rate across the six hospitals. The incidence rate was varied in sensitivity analysis.

### Vaccine efficacy

Vaccine efficacy in the target population is assumed to be 97.4% against pneumococcal meningitis and pneumococcal bacteraemia, 6% against all-cause pneumonia and 7% against otitis media in line with the Northern California Kaiser Permanente (NCKP) study [27]. Serotype coverage was adjusted to 66.7% based on the results of a local survey to account for potential differences in the serotype landscape in Malaysia relative to the NCKP trial [28].

Vaccination was assumed to be administrated in 4 doses at 2, 4, 6 and 12 to 15 months of age with efficacy commencing from the 1^st ^dose. PCV7 vaccination coverage is assumed at 90% of the birth cohort as the coverage of most vaccinations under the existing Malaysian national immunisation programme exceeds 95% [12]. A 100% compliance rate (i.e. that all infants in the cohort would to complete all 4 doses of vaccination) was assumed in order to reduce model complexity.

Consistent with previous economic studies on PCV7, the efficacy of PCV7 in the vaccinated birth cohort is assumed to decline by 1% per year up to the age of 5 years and by 3% per year from 6 to 10 years of age [22].

The effect of herd protection in the unvaccinated population is incorporated into the model by assuming reduction in the incidence of inpatient IPD by 32% in the 20-39 years group, 8% in the 40-64 years group and 18% in the ≥ 65 years group respectively [29]. The model assumptions on vaccine effects are described in Table 1.

### Outcomes estimation

Population data and life expectancy were based on 2006 population estimates for the various age cohorts in Malaysia. The life expectancy for infants was estimated to be 74.2 years [30] and the birth cohort was estimated to be 550,000 infants. The national population distribution by age group was 8,770,937 in the 20-39 years age group, 5,536,238 in the 40-64 years group and 1,051,371 in the greater than 65 years group [30]. Due to the absence of local age-specific mortality data, rates for paediatric and adult fatality were adopted from Singapore and Hong Kong respectively [31,32]. These assumptions are described in Table 1.

### Cost estimation

The cost of pneumococcal disease was determined from the perspective of the Malaysian healthcare system. These included the costs of direct medical resources including costs of physician consultation, all drugs consumed during admission and follow-up episodes, hospitalisation, diagnostic tests and surgical interventions. The treatment of an episode of pneumococcal disease covered the period from pre-admission care to hospitalisation and post-discharge out-patient follow-up. The costs of outpatient treatment that was not linked to any hospital admission were excluded.

Resources used in outpatient pre-admission and post-discharge treatment were estimated by the focus group discussion. The focus group delineated clinical pathways of treatment for pneumococcal disease which was then used to identify resources consumed to treat various clinical presentations.

Unit costs used in this analysis are shown in Table 3. These were obtained by a top-down costing approach to estimate cost of treatment in each hospital using the Clinical Costing Software Version 1.0 (CCM Ver. 1.0) developed by the Case-Mix Unit of the UKM Medical Centre. The software estimates unit costs for inpatient and outpatient treatment by distributing costs into three levels beginning with overhead cost centres, followed by intermediate cost centres and finally patient cost centres. Within each level, costs are distributed according to allocation factors such as floor size, staff counts, patient counts and inpatient days. The aforementioned focus group estimated that patient care in the Malaysian healthcare setting would typically have 2 to 3 visits to outpatient care prior to hospitalisation for pneumococcal disease. Therefore, depending on the disease manifestation, pre-admission and post-discharge outpatient treatment costs vary for an episode of hospitalisation as shown in Table 3.

**Table 3:** Model assumptions on costs

Cost Parameter	Value (RM)	Remarks
PCV7 vaccination cost (per infant)	888	4 dose regimen at current market price of RM222 per dose
**Pre-admission costs (adult and paediatric)**		
Meningitis, pneumonia and otitis media	206	Focus group discussion
Septicaemia	309	
**Post-discharge costs (adult and paediatric)**		
Meningitis, pneumonia and otitis media	309	Focus group discussion
Septicaemia	1,236	
**Paediatric cost per episode of hospitalised infection**		
Pneumococcal bacteraemia	7,076	
Pneumococcal meningitis	5,399	
Hospitalised all-Cause pneumonia	2,592	Weighted average cost of treatment for each episode of disease calculated using data from 6 local hospitals
Severe otitis media	2,554	
Weighted cost per episode of IPD	3,191	
**Adult cost per episode of hospitalised infection**		
Pneumococcal bacteraemia	6,141	
Pneumococcal meningitis	3,504	Weighted average cost of treatment for each episode of disease calculated using data from 6 local hospitals
Hospitalised all-Cause pneumonia	2,830	
Weighted cost per episode of IPD	3,768	
**Discount rate on costs and outcomes**	3%	
**Building use life**	20 years	
**Building depreciation**	5% p.a.	
**Instrument and vehicle use life**	5 years	
**Instrument and vehicle depreciation**	20% p.a.	

Note: Costs are measured in year 2007 Malaysian Ringgit (RM)

The following conventions were used for imputing capital and recurrent costs:

#### Capital Cost

Cost of buildings and fixtures have been included. The life span of buildings was estimated at 20 years with an annual depreciation of 5%, costs of instruments, transportation/vehicles have been determined at a life span of 5 years with an annual depreciation of 20%. Only areas and vehicles used within the activity scope of this study were considered.

#### Recurrent Cost

Recurrent costs included cost of labour and supplies. Labour costs included salaries, bonuses and allowances to healthcare personnel. Costs of supplies were calculated as the total cost of all purchases of medication and non-medication items. Utility costs due to water, electricity supply, telephone and waste maintenance were calculated for each activity within the scope of the study.

Both costs and benefits are discounted at 3% in the primary analysis. In sensitivity analysis costs were discounted at 3% while benefits were not discounted [33].

All costs were measured in 2007 Malaysian Ringgit (RM). The 2007 average exchange rates of US$1 to RM3.43 and €1 to RM4.71 were applied to currency conversions [34].

### Costs effectiveness analysis

A cost effectiveness model was used to estimate the impact of routine PCV7 vaccination of a birth cohort of 550,000 infants compared with no vaccination over a 10-year time horizon [22]. Model inputs included epidemiological and cost data from the six hospitals, focus group discussions and published sources as described above.

Cost-effectiveness was expressed as cost per life-year gained (LYG) where years of life are retained due to death avoided by means of an intervention, in this case universal infant vaccination. LYG was determined by taking into consideration average life expectancy minus the age of death due to IPD (LYG = Average life expectancy - age of expected death). Therefore, LYG was calculated by Malaysian life expectancy (74.2 years) minus the child or adult's age at the time of death due to disease. The incremental cost per life year gained was calculated by comparing routine infant vaccination with PCV7 against the alternative of no routine vaccination.

### Sensitivity analysis

Sensitivity analysis was conducted by reducing the cost from a four dose to three dose regimen. Based on results from other studies we assume no decline in efficacy from the dose reduction [35,36]. One way sensitivity analyses were also performed by varying the herd effects of vaccination [29], vaccine efficacy rates against paediatric cases of all-cause pneumonia and otitis media, and the serotype coverage of PCV7 [27,37]. Lastly, sensitivity analysis was performed by varying the discount rate on outcome to 0% (i.e. undiscounted outcome) while the maintaining a 3% discount on costs [33].

## Results

### Diseases incidence

Based on data from the six hospitals, the estimated age-specific incidence of hospitalised IPD, all-cause pneumonia and all-cause otitis media in Malaysia is as shown in Table 4. The highest rates of hospitalisation were observed for all cause pneumonia in the elderly above 65 years of age at 20.406 hospitalisations per 1000 population and in children under 4 years of age at 7.658 per 1000 population.

**Table 4:** Age specific incidence of pneumococcal disease by presentation

Clinical presentation	Annual incidence by age group and clinical presentation (per 1000 population ª)				
	0-4	5-19	20-39	40-64	≥ 65
Pneumococcal meningitis	0.347	0.057	0.035	0.027	0.046
Pneumococcal bacteraemia	0.463	0.1	0.152	0.593	2.533
Pneumococcal pneumonia	2.396	0.391	0.32	1.124	6.34
All-cause pneumonia	7.658	1.261	0.933	3.525	20.406
All-cause otitis media	0.491	0.337	0.152	0.294	0.165

ª Population data by age-group for each hospital catchment area for 2006-2007 was used as the denominator for calculation.

Source: Department of Statistics, Malaysia.

### Costs

At the proposed market price of RM222 per dose in a four dose regimen, a total vaccine cost of RM439.59 million would be incurred to vaccinate 495,000 infants, equivalent to 90% of the birth cohort of 550,000 as shown in Table 5. The direct medical cost of an episode of pneumococcal disease in a paediatric population varies from RM2,554 for an episode of hospitalised otitis media to RM7,076 for an episode of hospitalised pneumococcal septicaemia. The corresponding cost for adult care varies from RM2,830 for an episode of hospitalised all-cause pneumonia to RM6,141 for an episode of hospitalised pneumococcal septicaemia as shown in Table 3.

**Table 5:** Outcomes and cost effectiveness

	No vaccination	PCV7 vaccination	Incremental
**Hospitalised cases (n)**			
Total	83,430	73,845	-9,585
Paediatric	51,933	47,459	-4,474
Adult	31,497	26,386	-5,111
**Deaths (n)**			
Total	1,074	666	-408
Paediatric	639	303	-336
Adult	435	363	-72
**Life Years Lost (years)**			
Total	26,154.00	14,731.60	-11,422.50
Paediatric	19,482.60	9,239.90	-10,242.70
Adult	6,671.40	5,491.60	-1,179.80
**Costs (RM million)**			
Total	255.8	657.9	402
Vaccine	0	439.6	439.6
Hospitalisation episodes	255.8	218.3	-37.5
Paediatric	137.2	118.9	-18.3
Adult	118.7	99.4	-19.3
**ICER (per life year gained)**			RM35,196 (US$10,261)

### Outcomes

As shown in Table 5 over the 10-year time horizon of the model, vaccination of the birth cohort is estimated to prevent 4,474 cases of paediatric hospitalisations. These included 2,036 cases of IPD (consisting of 1,538 cases of bacteraemia and 498 meningitis), 2,289 cases of all-cause pneumonia and 149 cases of severe otitis media. Vaccination would also save 336 lives by reducing paediatric mortality from 639 to 303 deaths with 10,242.7 life years gained. With the addition of herd effects, a further 5,111 hospitalised cases of IPD and 72 deaths in adults would be avoided with 1,179.8 years of life saved.

### Cost effectiveness

Our model estimates that without vaccination, pneumococcal disease hospitalisations cost the health system approximately RM256 million (US$74.6 million) over 10 years. Routine infant vaccination will reduce hospitalisation costs by RM 37.5 million, to RM218.3 million over the same period.

Comparing routine infant vaccination with no vaccination over a 10 year time horizon, vaccination gains 11,422.5 life years at an additional cost of RM402 million. Thus, routine infant PCV7 vaccination incurs an incremental cost (ICER) of RM 35,196, (US$10,261) per life year gained (LYG) if herd protection in the adult population is included as shown in Table 5.

WHO guidelines on generalized cost-effectiveness analyses for public health interventions considers programmes to be cost effective when the cost-effectiveness ratio is between one and three times the country's GDP per capita [38]. As Malaysia's GDP per capita was RM23,920 (US$6,974) in 2007 [34], an ICER of RM 35,196 for routine PCV7 vaccination would be considered cost effective as it is well within Malaysia's cost effectiveness threshold of RM71,761 (US$20,922).

### Sensitivity Analyses

As shown in Table 6 the model ICER results were sensitive to variations in the number of doses of PCV7, the incidence rate of meningitis and septicaemia, and the efficacy of the vaccine against IPD. Varying the dose regimen to 3 doses results in ICER of RM25,576 per LYG. Varying other model parameters such as herd effects on the adult population, the vaccine serotype coverage, cohort vaccination rate, vaccine efficacy or disease incidence did not result in the ICER exceeding Malaysia's cost effectiveness threshold of RM71,761.

**Table 6:** Sensitivity analysis

Model parameters	Range of values	ICER (RM)
**Dose regimen**	3-4 doses	25,576 - 35,196
**Herd effects**		
Lower 95% bound on herd effects		
Age 20-30	23%	37,648
Age 40-64	1%	
Age ≥ 65	11%	
Higher herd effects		
Age 20-30	41%	31,886
Age 40-64	20%	
Age ≥ 65	31%	
**Discounting of costs (benefits not discounted)**	3%	33,140
**Vaccine serotype coverage**	61.7-71.7%	34,208 - 36,242
**% cohort vaccinated**	80-100%	31,711 - 32,751
**Vaccine efficacy**		
IPD	82.7% to 99.9%	34,377 - 40,896
Pneumonia	-1.5% to 11%	34,802 - 35,788
Otitis Media	4.1% to 9.7%	35,184 - 35,209
**Disease incidence**		
Meningitis		26,703 - 41.777
Bacteraemia	50% to 200% of base case	21,629 - 50,437
Pneumonia		34,723 - 35,433
Otitis Media		35,166 - 35,212

## Discussion

The results from the cost-effectiveness analysis show that routine infant vaccination with PCV7 can lead to a reduction in the incidence of all manifestations of pneumococcal disease and potentially save lives. In the paediatric population, universal vaccination is estimated to prevent 4,474 hospitalisations over a 10-year period. When the analysis included the impact of herd protection on the adult population, an additional 5,111 cases of hospitalised IPD and 72 deaths are estimated to be prevented.

At an ICER of RM 35,196 per LYG, the result of this study compares favourably against the WHO's recommended cost effectiveness threshold for Malaysia of RM71,761 as well as another large publically funded health programme in Malaysia that is the MOH chronic haemodialysis programme which has reported CER of RM33,642 in 2001 (equivalent to RM38,240 in 2007 after adjusting for general inflation) [39,34].

A meta-analysis by Isaacman examined the cost effectiveness of Prevenar vaccination in several countries as shown in Table 7[40]. Similar to the current study, the studies included were based on similar assumptions of 4 doses of vaccine administered, 10-year model timeline, 10-year vaccine protection, and cost and benefits discounted at 3% per year. They showed that the cost-effectiveness evidence for PCV7 supported the initiation of national immunization programs in countries considered. The cost effectiveness ratio of our study (at RM35,196 or € 7,479 per LYG) appears to be comparable to studies evaluated by Isaacman. However, such direct comparisons should be made cautiously as this present study has methodological differences to those considered in Isaacman's review. Specifically, the current study did not consider long-term cost savings from pneumococcal sequelae avoided and other outpatient cost savings.

**Table 7:** International comparisons of the cost-effectiveness of PCV7 (Source: Isaacman et al. 2008)

Author (Year), Country	**Total Vaccine Cost per dose**, €*	Cost-Effectiveness **(Payer perspective)**, €
McIntosh et al (2005)^Φ^ United Kingdom	€89.33	€4,767 per LYG
Melegaro and Edmunds (2004)^#^ United Kingdom	€72.55	€9,607 per LYG
Ray et al (2006)^§^ United States	€51.72	€14,750 per LYG
Wisløf et al (2006)^^^ Norway	€56.30	€159,503 per LYG

*Includes administration costs and costs of adverse events. Costs were converted to euros.

^Φ ^Costs were discounted 6%, benefits 0%

^# ^IPD rates were adjusted to reflect serotype distribution in the United Kingdom. Vaccine efficacy was 63% to 87% for the first 5 years. A lifetime analysis was performed, and a 3-dose schedule was used. Pneumococcus was responsible for 48% of pneumonia cases.

^§ ^Vaccine efficacy was 2.7% to 3.2% for simple otitis media, 14.4% to 16.9% for complex otitis media, and 59% to 72% for IPD. The indirect effect did not include the effect on pneumonia or otitis media.

^^ ^Indirect effects were modified to reflect serotypes in Norway: 8.9% in those aged 20 to 39 years; 12.9% in those aged 40 to 64 years; and 22.9% in those aged ≥ 65 years

The results of this study support the implementation of a nationwide programme of infant vaccination. We employed highly conservative incidence rates of pneumonia and otitis media in the paediatric population as only hospitalised (i.e. severe cases) were included in the model. The cost of pneumococcal disease treated exclusively in the outpatient setting was not included due to lack of reliable epidemiological and costing data. Our results do not estimate the true burden of disease seen in the community which are treated in the outpatient setting and therefore underestimates cost effectiveness. This can be regarded as a limitation since cost savings from reduction of pneumococcal disease treated in outpatient settings are likely to be considerable.

If the burden of disease in outpatients were also included in the model, the results would likely show greater cost effectiveness from outpatient pneumonia and otitis media cases prevented. Other potential effects of vaccination on related clinical presentations of pneumococcal disease such as sinusitis and septic arthritis were not included in the analysis. The analysis also did not consider the effects or cost consequences of long term sequelae (deafness, brain damage, focal neural deficit and chronic seizures) on paediatric survivors of pneumococcal meningitis.

We note that there were several other limitations to this study. Firstly, there was limited local data upon which to base estimates and thus assumptions based on international sources and clinician focus group discussion were necessary. This included the assumptions about hospital catchment and treatment populations. Secondly, the implementation of ICD coding varies across Malaysian hospitals affecting our ability to capture all cases through ICD codes alone. To avoid underestimating the pneumococcal disease incidence in Malaysia using ICD-10 only, patients were also identified by review of laboratory records.

Thirdly, the analysis excluded the programmatic costs that would be incurred in implementing a national vaccination programme. Finally, the model also assumes the indirect benefits in reduced hospitalisations are accrued from the vaccination of a single cohort only although it may be debatable if herd effects can be fully realized from vaccination of a single cohort only.

Reduction of antibiotic resistance is another factor favouring implementation of pneumococcal vaccination. Data from the U.S. suggests significant reductions in antibiotic prescribing for paediatric cases of otitis media have occurred following licensing of PCV7 [37]. Similarly, a reduction in the incidence of antibiotic-non-susceptible IPD has also been reported, and has been attributed to widespread PCV7 vaccination [41]. This potential benefit is of significant importance in Malaysia where *S. pneumoniae *isolates have exhibited increased antibiotic resistance.

Since this study was conducted, newer pneumococcal vaccines with wider serotype coverage such as PCV13 have since become available. Nevertheless, the analysis presented in this study shows clearly that even with the narrow serotype coverage from PCV7, routine infant vaccination is cost effective. Further analysis based on newer pneumococcal vaccines such as PCV13 would demonstrate even better cost effectiveness as the protection afforded from vaccination would be even greater than with PCV7.

## Conclusions

The results presented here show that universal infant vaccination with PCV7 has an incremental cost per life year gained of RM 35,196 (US$10,261) when only direct costs of hospitalisation and the impact of herd protection in the unvaccinated adult population are included. These cost savings from both the direct and indirect effects of vaccination show that a national infant vaccination programme with PCV7 is a cost-effective intervention for Malaysia. Thus, it can be considered a worthwhile investment for protection of the population against pneumococcal disease.

## Competing interests

The authors declare that they have no competing interests.

## Authors' contributions

SA conceived the study, participated in its design, participated in primary data collection, participated in project coordination and helped to draft the manuscript. GA participated in the design of the study, participated in primary data collection, conducted the epidemiological analysis and helped to draft the manuscript. ZA participated in primary data collection, participated in the epidemiological analysis and helped to draft the manuscript. SS participated in the participated in the design of the study, participated in primary data collection, participated in the epidemiological analysis and helped to draft the manuscript. AMN participated in primary data collection, performed the cost analysis and helped to draft the manuscript. AG participated in the analysis and helped to draft the manuscript. All authors have read and approved the final manuscript.

## Pre-publication history

The pre-publication history for this paper can be accessed here:

http://www.biomedcentral.com/1471-2334/11/248/prepub
